# A general framework for optimization of probes for gene expression microarray and its application to the fungus *Podospora anserina*

**DOI:** 10.1186/1756-0500-3-171

**Published:** 2010-06-18

**Authors:** Frédérique Bidard, Sandrine Imbeaud, Nancie Reymond, Olivier Lespinet, Philippe Silar, Corinne Clavé, Hervé Delacroix, Véronique Berteaux-Lecellier, Robert Debuchy

**Affiliations:** 1 Univ Paris-Sud 11, Institut de Génétique et Microbiologie UMR8621, F- 91405 Orsay, France; 2 CNRS, Institut de Génétique et Microbiologie, UMR8621, F-91404 Orsay, France; 3 Univ Paris-Sud 11, F- 91405 Orsay, France; 4 CNRS, Centre de Génétique Moléculaire, FRE3144, GODMAP, F-91190 Gif sur Yvette, France; 5 UFR des Sciences du Vivant, Université de Paris 7 - Denis Diderot, F-75205 Paris CEDEX 13, France; 6 Université Victor Segalen, Bordeaux 2, Institut de Biochimie et Génétique Cellulaires, UMR 5095, 1 rue Camille Saint Saëns, F-33077 Bordeaux cedex, France; 7 INSERM, Génomique Fonctionnelle des tumeurs solides, UMR U-674, IUH, Université Paris-Descartes, Paris, F-75010, France; 8 USR 3278 CNRS-EPHE CRIOBE-Université de Perpignan BP 1013 Papetoai Moorea 98729 Polynésie Française

## Abstract

**Background:**

The development of new microarray technologies makes custom long oligonucleotide arrays affordable for many experimental applications, notably gene expression analyses. Reliable results depend on probe design quality and selection. Probe design strategy should cope with the limited accuracy of *de novo *gene prediction programs, and annotation up-dating. We present a novel *in silico *procedure which addresses these issues and includes experimental screening, as an empirical approach is the best strategy to identify optimal probes in the *in silico *outcome.

**Findings:**

We used four criteria for *in silico *probe selection: cross-hybridization, hairpin stability, probe location relative to coding sequence end and intron position. This latter criterion is critical when exon-intron gene structure predictions for intron-rich genes are inaccurate. For each coding sequence (CDS), we selected a sub-set of four probes. These probes were included in a test microarray, which was used to evaluate the hybridization behavior of each probe. The best probe for each CDS was selected according to three experimental criteria: signal-to-noise ratio, signal reproducibility, and representative signal intensities. This procedure was applied for the development of a gene expression Agilent platform for the filamentous fungus *Podospora anserina *and the selection of a single 60-mer probe for each of the 10,556 *P. anserina *CDS.

**Conclusions:**

A reliable gene expression microarray version based on the Agilent 44K platform was developed with four spot replicates of each probe to increase statistical significance of analysis.

## Findings

Development of a gene expression microarray comprises several time-consuming and complex steps. Probe libraries are generated by commercial services or specialized design programs [1], which analyze nucleic acid physical parameters to identify probes that offer the best theoretical characteristics, in terms of specificity and sensitivity. Optimal probe design is a compromise between these two latter features, which are predicted by computational methods that assume probes are in solution, while arrays, in fact, consist of surface-immobilized probes. Therefore an empirical approach appears as the optimal strategy to assess the quality of the probe design outcome [2-4]. This experimental step has been long overlooked, due to microarray cost and reluctance to modify a fixed design. *In situ *synthesized oligomer arrays now offer great flexibility for changing probes, thus promoting the addition of real hybridizations in the probe selection process. Probe design should also take into account uncertainties of gene structure predictions [5,6] and genome databases re-annotations. Informatics tools allowing probe collection updating are available [7] but we are not aware of any established methods for dealing with potential annotation errors.

We chose medium length probes (60mers), which offer the best compromise between long oligonucleotide probes (50-80mers) prone to cross-hybridization [8,9] and short oligonucleotide probes (25-30mers) producing low signal intensity [10]. We used an ink-jet Agilent microarray platform and Agilent commercial service for designing probes. It delivers up to ten candidate probes per coding sequence (CDS). A single 60-mer probe can successfully detect gene expression at a low level [8]. We present computational and experimental processes to identify the optimal probe for each CDS.

### Computational selection of probes

The computational procedure selected a subset of four probes to be experimentally tested. The probe set was ranked automatically according to the following four criteria:

(1) Cross-hybridization capacity for non-target sequences. Each probe was aligned against the whole set of CDS using BLAST [11] with custom parameters (W = 7, z = 1 000 000, r = 2). These parameters were estimated from simulated data sets to detect a minimal identity of 70% on 20 contiguous bases [12]. A cross-hybridization identity (CHI) score was attributed to each probe, based on its identity with any non-target CDS (Table 1).

**Table 1:** Scores for *in silico *selection.

Criteria	Measure	Score values per criterion			
		
		0	1	4	20
CHI	% identity	60%	61- 84%	≥ 85%	NA^a^

Self-folding structure	ΔG	> -8 kcal/mol	≤ -8 kcal/mol	NA^a^	NA^a^

Probe position in CDS	Nucleotides numbered from CDS 3' end	1-500	500 - 1000	> 1000	NA^a^

Probe position relative to intron	Classes defined in Figure 1	Class 1	Class 2	Class 3	NA^a^

Sequence match	Used after genome re-annotation	Perfect match	NA^a^	NA^a^	Mismatch

^a ^Not Attributed

**Figure 1:**
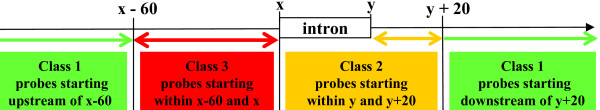
**Probe classes according to their position relative to an intron**. The black arrow represents the coding strand of a gene. Probes are identical to the coding strand. Nucleotide numbering begins at the first nucleotide of the contig, on the coding strand of the gene of interest; x represents the numbering of the last nucleotide of the exon preceding the 5' end of the intron, and y represents the numbering of the first nucleotide following the 3' end. Probe classes are indicated by the colored boxes.

(2) Thermodynamic properties and secondary structure stability. Secondary structures can compromise hybridization between the probe and its target. Possible hairpin structures were analyzed and the corresponding free energy (ΔG) was computed [13]. The parameters of the design program excluded probes with a low self-folding energy distribution, and therefore a high disqualifying score was not necessary (Table 1).

(3) Probe location relative to CDS 3' end. Labeling methods start from the polyA tail and become attenuated as the enzymes progress toward the 5' end [14]. Therefore, the selection procedure used gives the best scores to probes localized in the 3' end of the CDS (Table 1).

(4) Relative positions of probe and intron. It has been reported that only 15% of gene structures is predicted correctly across the coding region of some organisms [5]. Most probe design software does not select for probes according to their position relative to introns, whereas this criterion appears critical, notably for genomes with inaccurate intron prediction, often due to lack of ESTs. We therefore developed probe scores (Table 1) based on probe position relative to predicted introns (Figure 1). Probes that overlapped intron(s) were given a high score ensuring that they were rejected. The 3' boundaries of introns show little variations but the consensus is small [15] and prediction of intron 3' end is therefore uncertain. Consequently, probes located immediately adjacent to and downstream of the putative 3' end of introns were attributed a sub-optimal score.

A final score for the *in silico *quality of the probes was calculated from the sum of these four scores. A first round of selection identified probes with a final score below 4. If more than 4 probes were matched to a single CDS, we selected the four probes closest to the 3' end of the CDS. Probes that started within the last 100 nucleotides of a CDS were excluded to circumvent annotation uncertainties that are more frequent in the 3' region of CDS. For any CDS that have fewer than 4 probes, additional probes were selected by a second selection round that recovered probes overlapping intron(s) confirmed by EST(s), and allowing scores of up to 8. We excluded, however, probes that displayed a CHI of over 85% and probes that started upstream from the 3' terminal 1500 nucleotides of the CDS. A further probe-design stage was carried for CDS for which there was no, or only one, probe after the second selection round. For speed reasons, the probe design software ROSO [1,12] was used for this and subsequent designs, instead of Agilent commercial services. ROSO parameters are indicated in Additional file 1. Probes issued from this new design were submitted to the above *in silico *selection.

When genome re-annotation was released, probes were aligned against the updated set of CDS using BLAST [11] to identify probe-deficient CDS. New probes were then designed using ROSO [12] and the *in silico *scoring procedure was applied once again. Re-annotations also led to CDS modifications that resulted in mismatches with previously designed probes. These probes were attributed a score of 20 to ensure that they would be discarded from further analyses (Table 1).

### Experimental selection of probes

An experimental screening procedure was implemented to identify which one of the four *in silico *qualified probes measures 'true' gene expression with robust and consistent signal intensity. Different conditions, each with four biological replicates, were compared with a common reference, in an indirect design. The common reference was obtained by mixing RNA extracted from the different conditions under investigation. The three following criteria were used (formula and data used for calculation are indicated in Additional file 1):

(1) Signal-to-noise ratio. The determination of a signal-to-noise ratio (SNR) threshold is essential to distinguish a true signal from its background, and thus for the generation of high-quality microarray data. Subsequent data processing and biological interpretation of microarray results depend on the accuracy of this threshold. Two metrics were used to calculate the SNR values for each probe: (i) the signal-to-standard-deviation ratio (SSR) [16] and (ii) the signal-to-background ratio (SBR) [17]. SSR ratios greater than 10 are considered indicative of high quality arrays [16]. Probes with a SSR < 10 and a SBR < 2 for all samples or for all samples but one were discarded, as they possibly had a defective design.

(2) Signal reproducibility. The reproducibility of each probe is usually assessed with the normalized measure of signal dispersion for each probe by calculating the signal coefficient of variation (CV). As our experimental design consists exclusively of biological replicates, the CV measures biological heterogeneity, as well as technical variation causes. We minimized biological heterogeneity by using biological replicates with minimal genetic polymorphism ([18] and references therein). Lack of signal reproducibility, and the major source of variation (high CV), therefore, was attributable to probe defect. The threshold for CV was set at 0.75, to reject no more than approximately 1% of the total number of CDS. Probes with a CV > 0.75 for any condition were submitted to expert supervision to determine possible biological causes of heterogeneity and rejected if none was found.

(3) Signal intensity per CDS and per condition. We adapted the strategy of Paredes et al. [4], in which it was assumed that a probe targeted to a given CDS should have an optimal intensity signal that is similar to the average signal intensity of all probes targeted to this CDS. This rationale was applied to calculate two types of metrics: (i) Two median metrics were calculated from the normalized signal intensities obtained with the common reference RNA pool: the median of each probe targeted to a CDS (M_probe_) and the median for all probes targeted to this CDS (M_CDS_). Probes with M_probe _outside the interquartile range of M_CDS _were rejected. (ii) The average intensity (M_array_) of all probes targeted to a given CDS in each array and its 95% confidence interval (CI) were calculated from the normalized signal intensities obtained from hybridization with sample RNA. Probes were discarded if the signal intensity was outside M_array _± 1.5 CI for all arrays.

**Figure 2:**
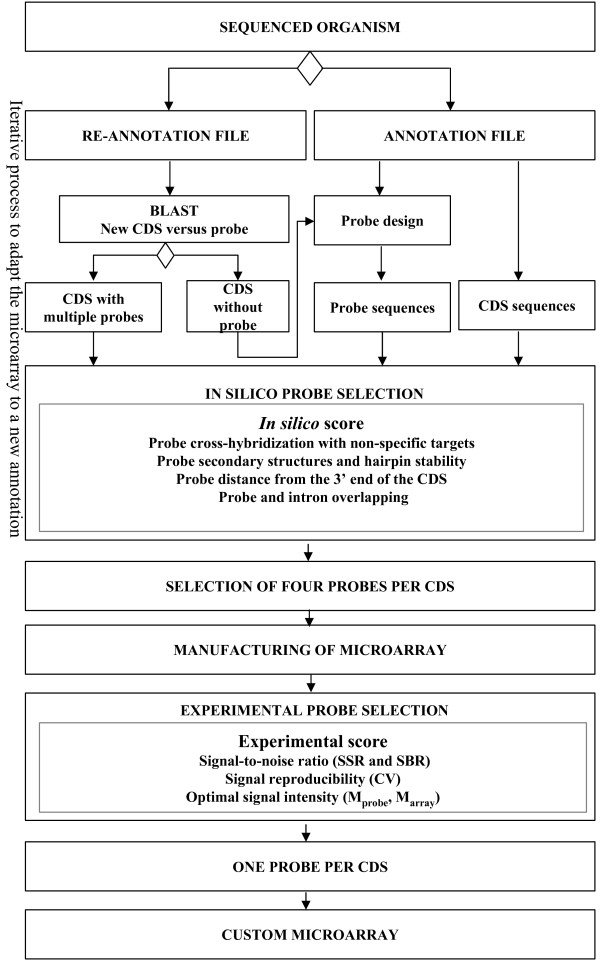
**Flow diagram for probe selection**.

Selected probes were pooled and the number of probes per CDS was determined. For CDS with more than one probe, the probe closest to the 3' end was selected as the final probe. The framework is depicted in Figure 2. Probe-deficient CDS were assigned one probe from the rejected probes set by expert-supervised selection.

### Application to *Podospora anserina*

The draft genome assembly of *Podospora anserina *contained 10,824 CDS when this work started (P. Silar and O. Lespinet, unpublished results) and was updated to 10,545 CDS [19] as work progressed. A total of 5,032 CDS have at least one intron but no EST to confirm intron position, emphasizing the value of selecting probes that do not overlap introns. Elimination of short and long CDS resulted in 10,539 CDS. The *in silico *ranking was reapplied resulting in 41,843 unique probes (Microarray v.2).

**Figure 3:**
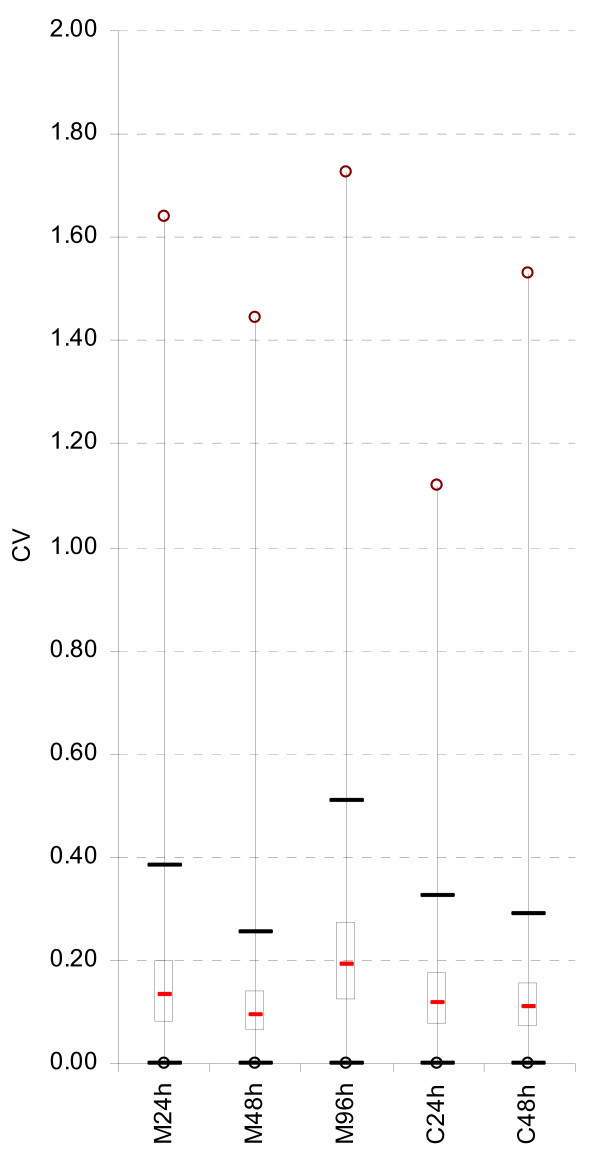
**Distribution of probe intensity CV in the five conditions used for the experimental validation of probes**. The distributions of probe intensity CV are presented in a series of five boxes (interquartile range) and whiskers plots. Hybridizations were performed on microarray v.2 with the cRNAs prepared from the five conditions (M24h, M48h, M96h, C24h, C48h) and labeled with Cy3. Each condition consisted of 4 biological replicates. The CVs were computed as indicated in Additional file 1. The median CV is 0.13, 0.10, 0.19, 0.12 and 0.11 for M24h, M48h, M96h, C24h and C48h, respectively.

**Figure 4:**
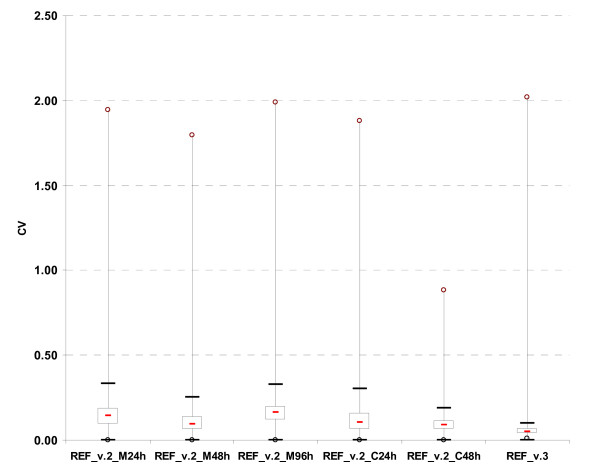
**Improvement of probe reproducibility in microarray v.3**. The distributions of probe intensity CV are presented in a series of six boxes (interquartile range) and whiskers plots. Hybridizations were performed with the reference common cRNA pool on microarray v.2 (5 hybridizations, REF_v.2_M24h, REF_v.2_M48h, REF_v.2_M96h, REF_v.2_C24h, REF_v.2_C48h, each with 4 technical replicates per probe) and v.3 (REF_v.3, 12 technical replicates per probe). The cRNAs were labelled with Cy5. The median CV is 0.14, 0.10, 0.16, 0.10, 0.09 and 0.05 for REF_v.2_M24h, REF_v.2_M48h, REF_v.2_M96h, REF_v.2_C24h, REF_v.2_C48h and REF_v.3, respectively.

Time courses of vegetative growth (24 h [M24h], 48 h [M48h] and 96 h [M96h]) and sexual crossing (24 h [C24h], 48 h [C48h] and 96 h [C96h] after fertilization) were used for extraction of RNA but only the M24h, M48h, M96h, C24h and C48h conditions were used for subsequent probe selection. Each condition had four biological replicates and including *mat + *and *mat - *strains [20], which were isogenic except at the mating-type locus. The common reference RNA pool was created by mixing equal amounts of RNA extracted from M48h, M96h, C24h, C48h and C96h. The materials and methods used for strains, cultures, nucleic acid extractions, RNA pool preparation and microarray analyses are described in Additional file 1. The numbers of outlier probes and probe-deficient CDS identified by experimental validation are shown in Table 2. As a result of low signal-to-noise ratio, 123 CDS had all of their probes rejected. These probes may either correspond to genes that were not expressed under the experimental conditions, or to false-positive genes resulting from over-annotation. The distribution of CV in the five experimental conditions is shown in Figure 3. Most of the probes (92%) rejected by this metric belong to M96h. Great transcription differences between *mat + *and *mat - *strains at M96h were characterized for some genes ([21] and unpublished observations); these differences are expected to persist in C24h and C48h conditions. Therefore, 27 probes (9 CDS) with a CV > 0.75 in two of the three above conditions were retained, as the high CV is likely biologically relevant. M_probe _and M_array _scores proved to be the most selective measures with 60% of probes being rejected after this analysis. At the end of the experimental validation, 9,822 CDS had at least one qualified probe. A total of 717 CDS were probe-deficient, because either one criterion, or a combination of criteria, was sufficient to eliminate all probes targeted to a given CDS (Table 2). For these CDS, one probe was chosen by supervised selection. The final array design contained 10,539 probes for nuclear CDS (Microarray v.3). As *P. anserina *is used as a model system for mitochondrial metabolism [22], 17 mitochondrial CDS probes were added to the final array. These probes underwent only the computational screening. Each array contained four replicates of each probe to improve statistical significance of results. Progression in microarray v.3 was confirmed by its signal CV which was lower than that obtained with v.2 upon self-to-self hybridization with the common reference cRNA pool (Figure 4). The median CV of microarray v.3 is similar to those obtained in the MAQC study with the commercial Agilent human microarray platform [23].

**Table 2:** Results of experimental scoring of probes.

Metrics	Rejected probes	Qualified probes	CDS with probes	Probe-deficient CDS^a^
SSR, SBR	2,013 (4.8%)	39,830 (95.2%)	10,327 (98.8%)	123 (1.2%)

CV	900 (2.1%)	40,943 (97.9%)	10,325 (95.2%)	125 (1.2%)

M_probe _and M_array_^b^	25,140 (60%)	16,480 (39.4%)	10,180 (97.4%)	140 (1.3%)

^a ^these CDS have had all of their probes rejected based only on one single metric. The combination of the three metrics resulted in more probe-deficient CDS (717) than the sum of probe-deficient CDS excluded because of a single criterion.

^b ^these metrics apply only to CDS with more than two probes. Therefore 223 probes, targeted to CDS with less than three probes, were excluded from this analysis.

The probe set is available at http://podospora.igmors.u-psud.fr/download.php. The data discussed in this publication have been deposited in NCBI's Gene Expression Omnibus [24] and are accessible through GEO Series accession number GSE20734 http://www.ncbi.nlm.nih.gov/geo/query/acc.cgi?acc=GSE20734. The final microarray is available from Agilent under the reference AMADID 018343.

## Competing interests

The authors declare that they have no competing interests.

## Authors' contributions

FB, SI and NR established the pipeline for probe scoring. FB and SI carried out RNA preparation, labelling and hybridization, and microarray analyses. NR designed probes with ROSO. OL participated to BLAST analyses and carried out probe mapping on the genome. FB, SI, NR, HD and RD prepared the manuscript. PS, CC, VBL, HD and RD conceived this study, collaborated to its design and coordination, and helped to draft the manuscript. The authors wish it to be known that, in their opinion, SI and NR should be regarded as joint Second Authors. All authors read and approved the final manuscript.

## Supplementary Material

Additional file 1 **Materials and methods**. Additional file descriptions text. Click here for file
