# Supplementary material for: A general framework for optimization of probes for gene expression microarray and its application to the fungus Podospora anserina
Source: BMC Res Notes. 2010 Jun 18;3:171. doi: 10.1186/1756-0500-3-171 (PMC2908635; doi:10.1186/1756-0500-3-171)
Supplement: Additional file 1 — Materials and methods. Additional file descriptions text. [file 1756-0500-3-171-S1.DOC]

### Materials and Methods (Bidard et al. )

**Parameters for ROSO [1] probe design.**

- Probes preferably located at the 3’ end of the CDS,

- The GC content was in the range of 35-55%,

- A probe should not have a contiguous single nucleotide repeat or a poly-(N) tract longer than six bases,

- The melting temperature (Tm) range was set between 68°C and 76°C.

**Number and type of conditions analysed by microarray and used for the calculation of metrics.**

Five conditions: M24h, M48h, M96h, C24h, C48h (see below).

Four biological replicates per condition.

One array per biological replicate, each array with four different probes per CDS (microarray v.2, see below).

**Calculation of signal-to-noise ratio.**

signal intensity = MedianSignal, raw median signal of feature in green or red channel (inlier pixel).

background intensity = BGUsed, background definition using spatial detrend and global background.

Signal intensity and background intensity were obtained with the Feature Extraction (FE, v9.5.3) software (Agilent) using the GE2-v4_95_Feb07.

Mean of signal intensities, mean and standard deviation of background intensities were calculated upon hybridization with cRNAs prepared from four biological replicates from one condition (Cy3 labelling). N = 4 values per probe.

Similar calculations were performed upon hybridization with the common reference cRNA pool (Cy5 labelling). N = 20 values per probe (five conditions, each condition with four biological replicates).

Six SSR values were obtained for each probe, each value corresponding to one condition (five conditions) and to the reference. Six SBR values were obtained for each probe from the same samples used for calculation of SSR values.

**Calculation of the coefficient of variation (CV).**

σ: standard deviation of normalized intensities was calculated upon hybridization with cRNAs prepared from the four biological replicates from one condition. N = 4 values per probe.

μ: mean of normalized intensities was calculated upon hybridization with cRNAs prepared from the four biological replicates from one condition. N = 4 values.per probe.

Distribution of CVs is indicated in Table 3.

#

# Table 3: Distribution of probe intensity CV in the five conditions used for the experimental validation of probes. RNA was labeled with Cy3 and hybridized on microarray v.2. The CVs were computed as indicated above.

**Calculation of signal intensity per CDS and per array.**

M_probe_ = m (REF normalized intensity) per probe, all arrays.

M_CDS_ = m (REF normalized intensity) per CDS, all arrays.

M_array_ = μ (condition normalized intensity) per CDS, one array.

REF normalized intensity = background substracted and Lowess adjusted intensity of signal obtained upon hybridization with the common reference cRNA pool. N= 20 values per probe (1 probe per array, 20 arrays) and 80 values per CDS (four different probes per CDS on each array, 20 arrays).

condition normalized intensity = background substracted and Lowess adjusted intensity of signal obtained upon hybridization with cRNA prepared from one condition. N = 4 values (four probes per CDS on one array).

***Podospora anserina* strains and growth conditions.**

| Condition name | Lifecycle phase | Culture description |
| --- | --- | --- |
| M24h | Early vegetative phase | *mat+* *S* strain harvested after 24 hours of growth on M2 media covered with a sheet of cellophane.  *mat-* *S* strain harvested after 24 hours of growth on M2 media covered with a sheet of cellophane. |
| M48h | Late vegetative phase/ early stationary phase | *mat+* *S* strain harvested after 48 hours of growth on M2 media covered with a sheet of cellophane.  *mat-* *S* strain harvested after 48 hours of growth on M2 media covered with a sheet of cellophane. |
| M96h | Stationary vegetative phase | *mat+* *S* strain harvested after 96 hours of growth on M2 media covered with a sheet of cellophane.  *mat-* *S* strain harvested after 96 hours of growth on M2 media covered with a sheet of cellophane. |
| C24h | Early sexual development | *mat+* *S* strain cultivated for 96 hours on M2 media covered with a cheesecloth, spermatization with *mat-* *S* spermatia and harvesting after 24 h.  *mat-* *S* strain cultivated for 96 hours on M2 media covered with a cheesecloth, spermatization with *mat+*  *S* spermatia and harvesting after 24 h. |
| C48h | Middle sexual development | *mat+* *S* strain cultivated for 96 hours on M2 media covered with a cheesecloth, spermatization with *mat-* *S* spermatia and harvesting after 48 h.  *mat-* *S* strain cultivated for 96 hours on M2 media covered with a cheesecloth, spermatization with *mat+*  *S* spermatia and harvesting after 48 h. |
| C96h | Late sexual development | *mat+* *S* strain cultivated for 96 hours on M2 media covered with a cheesecloth, spermatization with *mat-* *S* spermatia and harvesting after 96 h.  *mat-* *S* strain cultivated for 96 hours on M2 media covered with a cheesecloth, spermatization with *mat+*  *S* spermatia and harvesting after 96 h. |

**Table 4: *Podospora anserina* culture conditions for RNA extraction.**

*P. anserina* strains used in this study are the wild type *S* [2, 3] and *s* [3] strains of either *mat+* or *mat-* mating type. Growth was carried out on a minimal synthetic medium at 27°C as described by [4]. RNA was extracted from six different conditions described in Table 4. More information on the biology of *P. anserina* is available on <http://podospora.igmors.u-psud.fr/more.php>.

**Microarray versions.**

Three different microarray versions were designed on Agilent 44K (Agilent Technologies, Santa Clara, USA) and 4x44K-featured microarray, corresponding to successive steps of probe selection. Microarray v.1 includes one array *per* slide with 42,034 unique probes (three or four different probes per CDS corresponding to the draft genome annotation) and 2,256 standard control spots from Agilent. Microarray v.2 and v.3 consist of four arrays *per* slide; each array includes 1,417 standard control spots. Microarray v.2 contains 41,843 unique probes (1, 2, 3 or 4 different probes per CDS issued from the published release of genome annotation) while Microarray v.3 contains 10,556 unique probes replicated four times and randomly distributed on each array (42,224 features).

### RNA isolation and gene expression array hybridization.

For RNA extraction, strains were grown on a cellophane sheet (cat#1650193, BioRad, Marne la Coquette, France) or cheesecloth (Sefar Nitex 03-48/31, Dominique Dutscher, Brumath, France) placed on Petri-dishes. To produce a sufficient amount of biological material (20 to 100 mg), one to five Petri-dishes were inoculated simultaneously. Experimental growth conditions are described in Table 4. Biological material was scraped from cellophane sheets or cheesecloth and flash frozen with buffer RLT (Qiagen, Courtaboeuf, France) in liquid nitrogen. The biological material was ground with a Mikro-Dismembrator (Sartorius, Aubagne, France) in vessels frozen in liquid nitrogen. After removing cell debris by centrifugation, nucleic acids were passed through a Qiashreddrer (Qiagen) for shearing DNA and total RNA was purified on RNeasy Plant Mini Kit columns (Qiagen), with an additional DNase treatment. The quality and quantity of the total RNA was determined by using a NanoDrop ND-1000 spectrophotometer and the Bionalyzer 2100 system (Agilent) as described in [5].

The common reference RNA pool consisted of a mixture with identical quantities of total RNA extracted from M48h, M96h, C24h, C48h and C96h (Table 4).

For transcriptome microarray experiments, target preparation, hybridization and washing were done following the two-color microarray-based gene expression analysis instructions (version 5.0, February 2007) as described by the manufacturer (Agilent). The number of replicates is indicated in Table 5. One microgram aliquots of total RNA were amplified and Cy-labelled with the Agilent’s Low RNA input fluorescent linear amplification (LRILAK) PLUS kit (Agilent) along with Agilent's Two-Color RNA Spike-in Kit. The labelling efficiency and the product integrity were checked as described by [5]; then, 750 ng (Microarray v.1) and 825 ng (Microarray v.2 and v.3) of each of the Cy3- and Cy5-labeled targets were mixed and incubated on an Agilent microarray slide for 17 hours at 65°C, in a rotating oven (6 rpm for a 1x44K and 10 rpm for a 4x44K array format), using an Agilent *in situ* hybridization kit. The slides were washed and water was removed by centrifugation at 800 rpm for 1 min.

| Conditions | Number of biological replicates | Number of replicates of mat+ mating type | Number of replicates of *mat-* mating type |
| --- | --- | --- | --- |
| M24h | 4 | 2 | 2 |
| M48h | 4 | 2 | 2 |
| M96h | 4 | 2 | 2 |
| C24h | 4 | 2 mat+ female strains | 2 mat- female strains |
| C48h | 4 | 2 mat+ female strains | 2 mat- female strains |

**Table 5: Number and type of replicates for microarray hybridization.**

### Microarray data acquisition, processing and analysis

Microarrays were scanned using the Agilent DNA microarray Scanner (Agilent) at 5 micron resolution using the extended dynamic range (XDR) feature. Spot and background intensities were extracted with the Feature Extraction (FE, v9.5.3) software (Agilent) using the GE2-v4_95_Feb07 default protocol. Preliminary array quality was assessed through the use of Agilent control features as well as spike-in controls (Agilent 2-Color Spike-in Kit for RNA experiment). Subsequent flagging was done according to the GenePix Pro software (Molecular Devices Sunnyvale, CA, USA) nomenclature, including four levels of flags (good [100], bad [-100], not found [-50], moderate [0]). FE software raw data from arrays issued of Microarray v.1 platform were processed with the MAnGO software [6] with no background subtraction and the Robustpline Lowess normalization method [7]. FE software normalized data (Local background was subtracted and Lowess normalization) from arrays issued of Microarray v.2 and v.3 were processed with MAnGO [6]. Moderate *t*-test with adjustment of *p*-values [8] was computed to measure the significance with each expression difference.

Microarray experiment was done by reversing (Microarray v.1) or not (Microarray v.2 and v.3) dye hybridization for experimental and reference samples. Two (Microarray v.1) or four (Microarray v.2 and v.3) biological replicates were used.

**References.**

1. Reymond N, Charles H, Duret L, Calevro F, Beslon G, Fayard JM: **ROSO: optimizing oligonucleotide probes for microarrays**. *Bioinformatics (Oxford, England)* 2004, **20**:271-273.

2. Espagne E, Lespinet O, Malagnac F, Da Silva C, Jaillon O, Porcel BM, Couloux A, Aury JM, Segurens B, Poulain J *et al*: **The genome sequence of the model ascomycete fungus *Podospora anserina***. *Genome biology* 2008, **9**:R77.

3. Rizet G: **Les phénomènes de barrage chez *Podospora anserina.* I. Analyse génétique des barrages entre souches *S* and *s*.** *Rev Cytol Biol Veg* 1952, **13**:51-92.

4. Esser K: **The genetics of *Podospora anserina***. In *Handbook of Genetics*

*Volume 1*. Edited by King RC. New York: Plenum Press; 1974:531-551.

5. Imbeaud S, Graudens E, Boulanger V, Barlet X, Zaborski P, Eveno E, Mueller O, Schroeder A, Auffray C: **Towards standardization of RNA quality assessment using user-independent classifiers of microcapillary electrophoresis traces**. *Nucleic acids research* 2005, **33**:e56.

6. Marisa L, Ichante JL, Reymond N, Aggerbeck L, Delacroix H, Mucchielli-Giorgi MH: **MAnGO: an interactive R-based tool for two-colour microarray analysis**. *Bioinformatics (Oxford, England)* 2007, **23**:2339-2341.

7. Smyth GK, Speed T: **Normalization of cDNA microarray data**. *Methods (San Diego, Calif* 2003, **31**(4):265-273.

8. Benjamini Y, Hochberg Y: **Controlling the false discovery rate: a practical and powerful approach to multiple testing.** *J Roy Stat Soc Ser B (Statistical Methodology)* 1995, **57**(1):289-300.
